# Comparative effects of tannic acid and ZnO nanoparticles on hydration, microstructure, and antimicrobial performance of Portland cement

**DOI:** 10.1039/d6ra03950b

**Published:** 2026-07-03

**Authors:** Alaa Mohsen, M. S. Amin, Hany M. Abd El-Lateef, Mai M. Khalaf, Hassan H. Hammud, Saad Shaaban, Nagih M. Shaalan, Musa A. Said, Abdullah A. Saber, M. Ramadan

**Affiliations:** a Faculty of Engineering, Ain Shams University Cairo Egypt; b Chemistry Department, College of Science, Taibah University P.O. Box 344 Al-Madinah Al-Munawwarah Saudi Arabia; c Chemistry Department, Faculty of Science, Ain Shams University Cairo Egypt m.ramadan@sci.asu.edu.eg; d Department of Chemistry, College of Science, King Faisal University Al-Ahsa 31982 Saudi Arabia hmahmed@kfu.edu.sa; e Department of Physics, College of Science, King Faisal University Al-Ahsa 31982 Saudi Arabia; f Department of Chemistry, Faculty of Science, Islamic University of Madinah Madinah 42351 Saudi Arabia; g Department of Biology, College of Science, Imam Mohammad Ibn Saud Islamic University (IMSIU) Riyadh 11623 Saudi Arabia

## Abstract

Despite the well-documented antimicrobial performance of ZnO nanoparticles, their incorporation into Ordinary Portland cement (OPC) is often accompanied by unfavorable effects, including delayed setting and deterioration of early-age mechanical properties. This investigation evaluates tannic acid (TA), a bio-derived organic compound, as a sustainable alternative cement additive to nano-ZnO. OPC pastes were modified with varying dosages of TA or nano-ZnO, and their influence on setting characteristics, workability, hydration progress, chemically combined water, free lime content, compressive strength, pore structure, phase composition, and microstructural development was systematically investigated. The results show that TA accelerates hydration at low dosage (0.25%) but exhibits a retarding effect at higher content (1%), whereas nano-ZnO consistently retards hydration regardless of concentration. At 3 days of curing, OPC containing 0.25% nano-ZnO attained a compressive strength of 78.3 MPa, while a pronounced reduction to 7 MPa was observed at 1% addition. In contrast, TA-modified systems demonstrated more stable mechanical performance, achieving 68.3 MPa at 0.25% and maintaining approximately 50 MPa at 1%. BET/BJH pore structure analysis revealed that both additives at 0.25% induced significant pore refinement, reducing the mean pore diameter from 67.92 nm to 48.52 nm for TA and 45.57 nm for nano-ZnO. Mineralogical and microstructural analyses (XRD, FTIR, SEM/EDX) indicated that TA promotes silicate dissolution and enhances the formation of portlandite, stratlingite, and tobermorite-like CSH phases. Conversely, nano-ZnO was found to inhibit hydration through the formation of Zn(OH)_2_ surface layers and calcium zincate hydrates (CZH). Antimicrobial tests confirmed effective inhibition of *E. coli*, *E. faecalis*, and *C. albicans* for both additives. The findings suggest that tannic acid provides improved hydration compatibility and mechanical reliability while retaining antimicrobial functionality, making it a viable alternative additive for cementitious systems.

## Abstract 2

A low dosage of tannic acid promoted the hydration reaction, refined pore structure, enhanced early strength, and developed antimicrobial performance of Portland cement.

## Graphical abstract

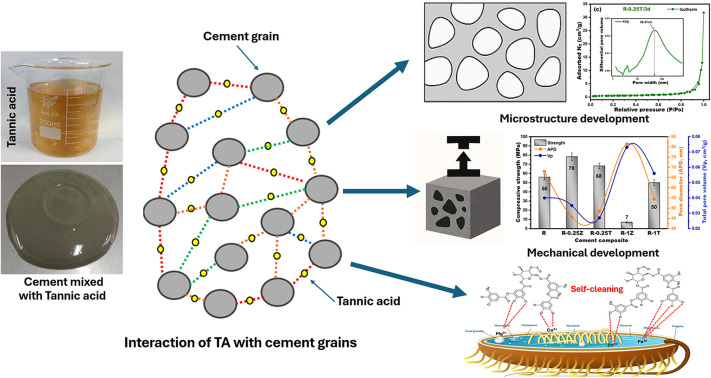

## 1. Introduction

Mineral-based construction materials such as concrete, mortar, plaster, and bricks are inherently porous and often contain microstructural defects like pits and cracks. These imperfections create favorable conditions for microbial colonization, including fungi, bacteria, and algae. The growth of such organisms is not only unsightly but also poses serious health risks, particularly from mold exposure, which can lead to respiratory conditions such as asthma, rhinitis, and mycotoxicosis.^1^ In addition to health concerns, microbial activity accelerates material degradation. Microorganisms secrete organic acids such as gluconic, ascorbic, lactic, formic, citric, oxalic, and fumaric that chemically interact with the mineral components of the materials. These acids enhance the dissolution and leaching of structural elements, compromising the integrity of the construction. Over time, this biochemical deterioration can result in significant structural damage and increased maintenance costs.^2,3^

In response to these challenges, the development of self-cleaning and antimicrobial construction materials has emerged as a critical area of research. One widely adopted strategy involves the incorporation of nanoparticles, particularly those composed of heavy metal oxides, into the matrix of construction materials.^4–6^ These nanoparticles exhibit unique surface and electronic properties, including specific band-gap energies, which enable them to act as photocatalysts.^7^ When exposed to visible or ultraviolet light, these materials absorb the radiation and initiate the radiolysis of water molecules on their surfaces. This process generates reactive oxygen species (ROS), such as hydroxyl radicals and superoxide anions, which are highly effective in disrupting microbial cell membranes and inhibiting biofilm formation.^8,9^ Among the various nanoparticles explored for cementitious applications, titanium dioxide,^6^ silver,^10^ and zinc oxide NPs^11^ have shown promising multifunctional properties. Titania and Ag nanoparticles are valued for their self-cleaning, antimicrobial, and durability-enhancing effects. However, their use in cement systems is not without limitations. TiO_2_ relies on ultraviolet (UV) light for activation and tends to agglomerate, which can hinder its dispersion and reduce effectiveness in indoor environments.^12^ Silver nanoparticles, while highly effective against microbial growth, are expensive and pose potential environmental and health risks due to the leaching of toxic silver ions.^13^

Nano zinc oxide has attracted attention for its antimicrobial properties; however, its application in cement-based materials remains limited due to certain mechanical and hydration-related challenges.^14^ Numerous studies have demonstrated that incorporating small dosages of nano zinc oxide into cement and geopolymer pastes significantly enhances their resistance to a wide range of microbial strains. These include various fungi such as *Aspergillus fumigatus* (ATCC 26933), *Aspergillus niger* (ATCC 1015, MTCC 1344), *Aspergillus terreus* (ATCC 20542), *Mucor circinelloides* (AUMMC 11656), and *Penicillium glabrum* (OP69417),^9,15^ as well as bacterial species like *Bacillus subtilis* (ATCC 6633), *Salmonella typhi* (ATCC 6539), *Pseudomonas aeruginosa* (ATCC 90274), *Escherichia coli* (MTCC 1652), and *Staphylococcus aureus* (MTCC 96).^11,16^ On the other hand, several studies have reported that nano-ZnO alters the early hydration kinetics of cement. Specifically, the formation of zinc hydroxide and layered calcium-zincate-hydrates during the hydration of calcium silicates has been shown to impede water transport, thereby delaying the hydration process. For instance, Klapiszewska *et al.*^17^ found that 0.1 wt% nano-ZnO extended the induction period from 2.5 to 6.3 hours and lowered hydration heat. Mohsen *et al.*^11^ observed that incorporating 1% ZnO nanoparticles into cementitious pastes significantly delayed the hydration process, extending the setting time to 512 minutes well beyond the standard limits for cementing materials. Liu *et al.*^18^ reported that 2 wt% nano-ZnO significantly delayed OPC setting times by over 250% and reduced 1 day strength by ∼80%. Zailan *et al.*^19^ observed that 2.5–7.5 wt% nano-ZnO reduced the 28 days compressive strength of cementing composite by up to 54.3%. Despite these drawbacks at higher concentrations, some investigations have demonstrated that lower dosages (0.05–0.5) may offer performance benefits.^14,20^

Tannic acid (TA) was selected as a natural cementing admixture to test our hypothesis due to its favorable properties. As a naturally occurring polyphenol abundantly present in various plant tissues, TA is recognized for its sustainability, environmental friendliness, and low cost.^21,22^ With a molecular formula of C_76_H_52_O_46_, its structure is rich in catechol groups, which confer a strong binding affinity to diverse surfaces through both covalent and non-covalent interactions, such as hydrogen bonding.^23^ Recent research has shown that TA can form hydrogen bonds with siloxane sites, facilitating robust adhesion to concrete substrates. These interactions suggest that TA can enhance the mechanical properties of cement-based materials by cross-linking^24^ with hydration products like calcium silicate hydrates (CSHs) and calcium aluminosilicate hydrates (CASHs).^25,26^

TA can interact with cementitious materials *via* strong hydrogen bonds, calcium chelation, and electrostatic interactions (π–π stacking) which are critical in the early stages of cement hydration. These interactions can lead to the formation of calcium tannate complexes^27^ that act as nucleation sites, thereby accelerating the precipitation of hydration products such as CSHs, CASHs and CAHs. However, depending on its concentration and the specific cement formulation, TA may also delay setting by temporarily sequestering calcium ions, thus slowing the supersaturation required for initial hydrate formation. These dual effects suggest that TA's impact on setting time is dose-dependent and closely tied to its chemical interactions within the cementitious system. Previous investigations have demonstrated that tannic acid (TA) can significantly delay the setting of cement by interfering with early hydration.^28^ TA binds strongly to calcium ions, forming calcium tannate nanoparticles, which reduces the availability of free calcium needed for hydration and pozzolanic reactions. This interaction results in a notable extension of both the initial and final setting times of cement pastes.^29^ On the contrary, other studies confirmed that tannic acid has been shown to enhance the dissolution of aluminum precursors.^30^ The released aluminum ions readily convert to Al(OH)_3_ within the cementitious system. This transformation contributes to an accelerated setting process by promoting the early formation of solid phases.^31^ Furthermore, Al(OH)_3_ can serve as a precursor in the synthesis of ettringite and monosulfate, which are integral to early strength development. However, excessive formation of Al(OH)_3_ may lead to undesirable effects, including flash setting and disruption of the uniform development of other hydration products, potentially compromising the performance of the cement paste.^9,32^ Kharouf *et al.*^33^ concluded that TA accelerates the setting process of mineral trioxide aggregate (MTA) cements while simultaneously enhancing both their surface characteristics and overall bulk properties. Qian *et al.*^34^ employed a high concentration of TA to improve the dispersion of metakaolin and enhance the workability of ordinary Portland cement-metakaolin (OPC-MK) blends. This improvement was attributed to the electrostatic repulsion and steric hindrance effects imparted by the polyphenolic groups of TA. Additionally, the incorporation of TA led to a significant increase in 28 days compressive strength, which was associated with the formation of a highly densified calcium silicate hydrate (CSH) matrix.

Thanks to catechol groups, TA exhibits broad-spectrum antimicrobial activity, demonstrating efficacy against a variety of fungal and bacterial strains.^35^ Its antifungal properties have been particularly noted against clinically relevant Candida species, including *C. albicans*, *C. auris*, *C. glabrata*, *C. parapsilosis*, and *C. tropicalis*.^36^ TA inhibits fungal growth by disrupting membrane integrity, suppressing hyphal development, and reducing biofilm formation and protease secretion.^37^ In addition to its antifungal action, TA has shown potent antibacterial activity against both Gram-positive and Gram-negative bacteria, such as *Staphylococcus aureus*, *Escherichia coli*, *Klebsiella pneumoniae*, and *Pseudomonas aeruginosa*. Its mechanism involves compromising cell wall integrity and interfering with microbial adhesion and colonization.^35,38^

Although tannic acid and other bio-based additives have been previously reported in cementitious systems, their direct comparison with conventional antimicrobial nanoparticles remains limited. In this work, a systematic and integrated comparative framework is adopted to evaluate tannic acid and nano-ZnO as functional additives for OPC at identical dosages (0.25, 0.5, and 1 wt%). The novelty of this work lies in combining a multidimensional assessment of setting times, workability, hydration behavior, pore structure, and mechanical performance (1, 3, and 28 days) with advanced microstructural characterization techniques (XRD, FTIR, BET/BJH, and SEM/EDX). This approach enables direct correlation between strength development, microstructural evolution, and the immobilization behavior of additives within hydration products. Furthermore, the report incorporates a detailed evaluation of the leaching behavior of both additives, using Atomic Absorption Spectroscopy for Zn^2+^ and UV-vis spectrophotometry for tannic acid, providing new insight into their stability, retention efficiency, and potential environmental impact. In addition, statistical analysis is employed to improve result reliability and enable quantitative comparison between different formulations.

## 2. Materials and testing

### 2.1. Materials

Ordinary-Portland cement (OPC), nano-ZnO and tannic acid (TA) were the materials used to prepare the cementitious system. OPC with a type CEM I (42.5 N) was supplied from National Company for Cement, Beni Seuf, Egypt. Its Blaine surface area was measured by the Air Permeability Tester (APT, HUMBOLDT, model: H3056.3F); it was found to be 3800 cm^2^ g^−1^. Also, its mineral-oxide composition was obtained using X-ray fluorescence (XRF, Xios, model: PW-1400), as illustrated in Table 1.

**Table 1:** Chemical oxide compositions for OPC (mass, %)

Material	SiO_2_	Al_2_O_3_	Fe_2_O_3_	CaO	MgO	SO_3_	Na_2_O	K_2_O	Cl^−^	LOI
OPC	20.69	4.67	3.51	64.11	1.27	2.92	0.29	0.22	0.02	2.30

**Fig. 1:**
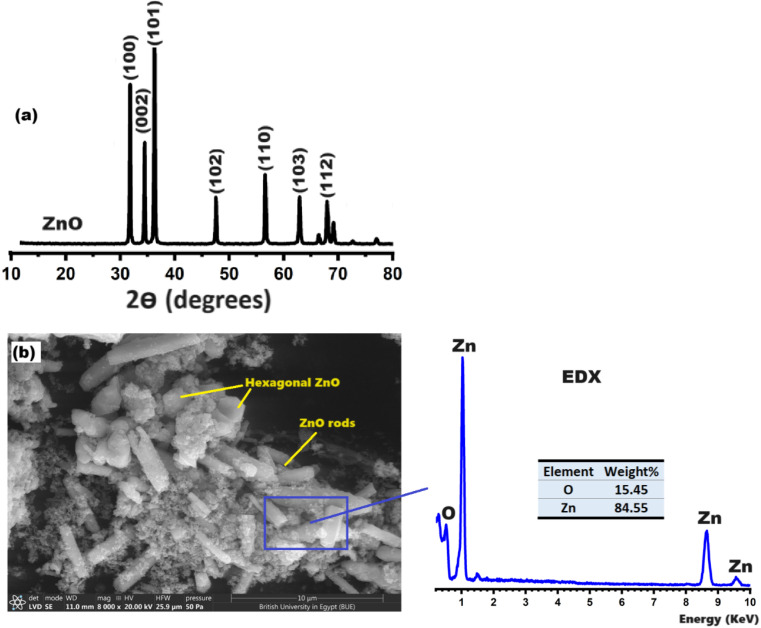
Characterization of nano-ZnO (a) XRD and (b) SEM/EDX.

The nano-ZnO was synthesized *via* the Co-precipitation method using zinc nitrate (Zn(NO_3_)_2_) as a zinc precursor, cetyl-trimethyl-ammonium bromide (CTAB) as a surfactant and ammonium hydroxide (NH_4_OH) as a precipitating agent. The Zn(NO_3_)_2_, CTAB and NH_4_OH were delivered from El-Nasr Company, Egypt. The synthesis procedure includes preparing 0.5 M Zn(NO_3_)_2_ solution, 0.3 M CTAB and 3 M NH_4_OH solution. Firstly, 1 L of Zn(NO_3_)_2_ solution was mixed with 50 mL of CTAB for 1 h. After that, NH_4_OH solution was added dropwise to the mixture till reaching a pH of 7–8; then the mixing was continued for another 1 h. The formed gel (Zn(OH)_2_) was left for 24 h, then filtered, washed several times with distilled water, dried and calcined at 500 °C for 2 h.^11,39^ The prepared nano-ZnO was characterized through identifying the phase composition using X-ray diffraction (XRD, Malvern-PANalytical, model: X'Pert-Pro), examining the morphology using a scanning-electron microscope with X-ray analyzer (SEM/EDX, Thermo-Scientific, model: Quatro-S) and measuring the surface area by the Brunauer–Emmett–Teller model (BET) using an automatic surface-area and pore-size analyzer (N_2_-adsorption–desorption, MICROTRAC, model: BELSORP® MINI X). The XRD-pattern (Fig. 1a) indicates the preparation of pure nano-ZnO through the existence of sharp peaks related to the wurtzite phase (ZnO, 2*θ* = 31.71, 34.43, 36.25, 47.54, 56.61, 62.87 and 67.95° with reflections of 100, 002, 101, 102, 110, 103 and 112, respectively, PDF# 01-071-6424). The crystallite size was measured using the Debye–Scherrer model, and it was found that the average size is 45.72 nm. The SEM-micrograph (Fig. 1b) demonstrated that nano-ZnO has distinct rods intermixed with hexagonal shapes and fine spheroids; Furthermore, the EDX-analysis shows that Zn and O wt% are 84.55 and 14.45, referring to the high purity. The surface area was found to be 29 900 cm^2^ g^−1^.

**Fig. 2:**
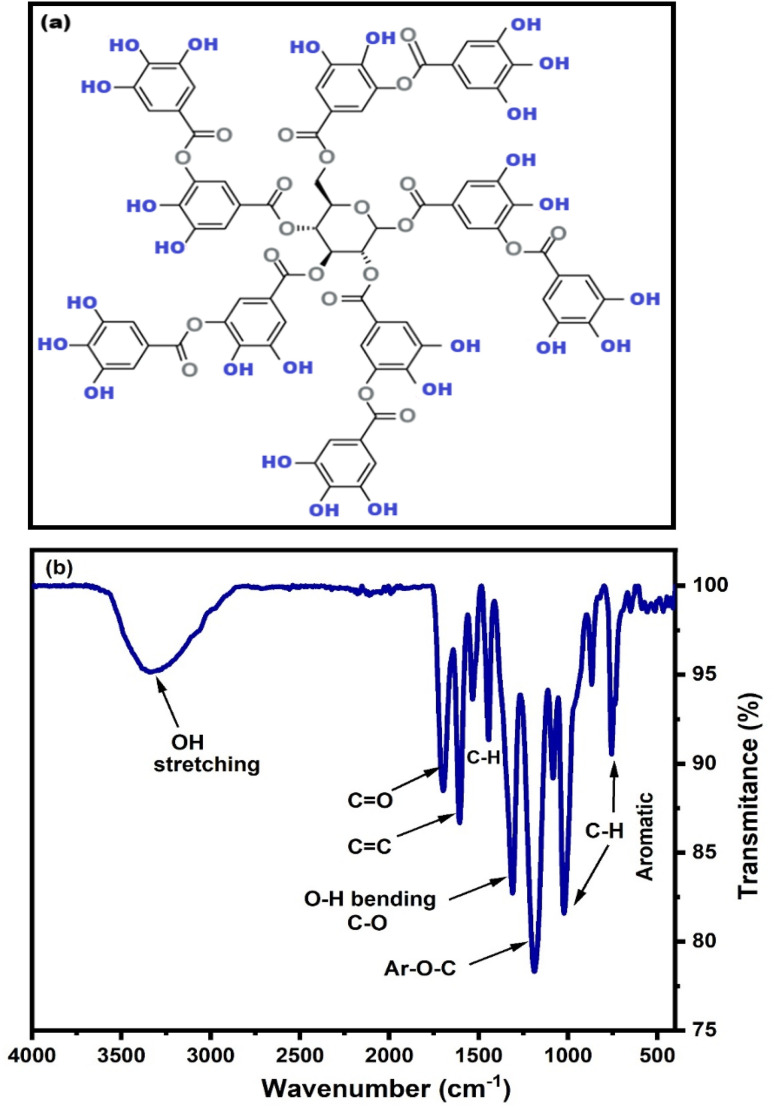
Characterization of tannic acid (a) molecular structure and (b) FTIR-spectroscopy.

Finally, TA, as a natural admixture, was purchased from the British Drug Houses Ltd (BDH), England. The functional groups in TA were examined using Fourier-transform infrared spectroscopy (FTIR, Mattson-1000, model: Unicam) in the range of 400–4000 cm^−1^. As shown in Fig. 2a, tannic acid's molecular structure comprises multiple functional groups, which are distinctly observable through FTIR analysis, as depicted in Fig. 2b. The most prominent feature is a broad O–H stretching band in the 2850–3600 cm^−1^ range, indicative of extensive hydrogen bonding due to its multiple hydroxyl groups. A well-defined C=O stretching peak between 1650–1730 cm^−1^ suggests the presence of ester or carboxylic acid groups. A sharp absorption at 1610 cm^−1^ corresponds to aromatic C=C stretching, confirming the compound's polyphenolic structure. Additional characteristic bands at 1315, 1190, 1020, and 755 cm^−1^ are attributed to C–O stretching, O–H bending, aromatic C–O–C stretching, and aromatic C–H bending, respectively.^40^ These spectral features collectively highlight the complex and multifunctional chemical nature of tannic acid.

### 2.2. Specimens' preparation and testing

**Fig. 3:**
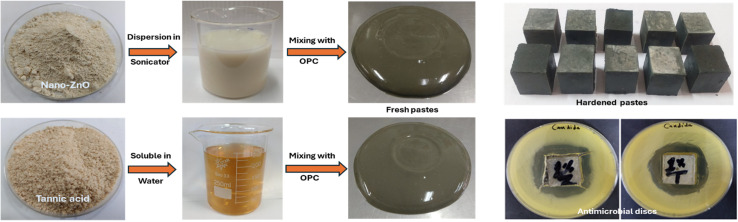
Real images of modified cement pastes containing nano-ZnO and tannic acid.

Seven mixes were prepared in this study, as clarified in Table 2. One of them is a reference (coded: R) prepared from mixing OPC with water (water/cement (*W*/*C*) ratio equal to 0.27) in a Hobert mixer for 5 min. The other six mixes were modified with different dosages (0.25, 0.5 and 1 wt%) of nano-ZnO (coded: R-0.25Z, R-0.5Z and R-1Z, respectively) or TA (coded: R-0.25T, R-0.5T and R-1T, respectively). In these mixes, the same *W*/*C* ratio (0.27) was used as in the R specimen. In the case of the inclusion of nano-ZnO, the ultrasonic sonicatior was used to improve its dispersion. The nano-ZnO was mixed with water and stirred in a mechanical stirrer for 5 min until completely wetted. After that, the solution was sonicated in an ultrasonic homogenizer (Labtron, model: LUHS-A12, 650 W and 25 kHz) for 15 min, then mixed with OPC as illustrated above. Regarding the specimen containing TA, it is first dissolved in the mixing water and then mixed with OPC. Fig. 3 shows a real image of solutions containing dispersed nano-ZnO and dissolved tannic acid, as well as fresh and hardened pastes.

**Table 2:** Mix design of the prepared pastes (mass, %)

Composite	OPC %	Nano-ZnO (%)	Tannic-acid %	*W*/*C* ratio
R	100	—	—	0.27
R-0.25Z	100	0.25	—	0.27
R-0.5Z	100	0.5	—	0.27
R-1Z	100	1	—	0.27
R-0.25T	100		0.25	0.27
R-0.5T	100		0.5	0.27
R-1T	100		1	0.27

The impact of nano-ZnO and TA on the fresh characteristics of cement pastes was evaluated *via* measuring (i) the workability using a mini-slump test using Abram's cone. In this test, a high *W*/*C* ratio of 0.45 was used to allow the observation of the variations in the spread-area;^41–43^ and (ii) the setting time using the Vicat apparatus according to ASTM C191-19.^44^ Both the spread area and the setting time were determined in triplicate for each sample. Regarding the mechanical properties, the fresh pastes were cast in a 2.5 × 2.5 × 2.5 cm stainless-steel cubic mold. The curing was performed in a humidity chamber at 98 ± 2% relative humidity and 25 °C for 24 h, then demolded and cured was continued under water till testing (1, 3 and 28 days). For all specimens at each time, the average compressive-strength value of three samples was measured using a compression machine (Control, Max-load of 250 kN) according to ASTM C109M-20b.^45^ To verify the data of fresh and hardened properties, some specimens were selected for further analysis using XRD, FTIR, BET/BJH and SEM/EDX to monitor the changes in the phase composition of hydration products, texture characteristics and microstructure of the cement system before and after inclusion of nano-ZnO and TA.

The hydration kinetics were evaluated by measuring chemically combined water content (*W*_*n*_, %) and the free lime content (CaO, %) of the hardened samples at 1, 3, and 28 days of hydration. Approximately 1 g from each of two dried specimens was placed in porcelain crucibles, ignited for one hour at 1000 °C in a controllable muffle furnace, then cooled in a desiccator and reweighed. The non-evaporable water, representing the water chemically bound within the hydration products, was calculated using the following equation:1
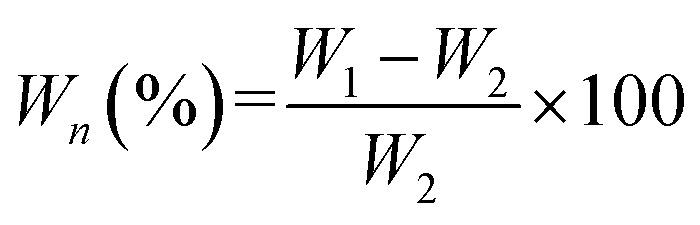


*W*_1_: it refers to the weight of the sample in its dried state before the ignition step (g).

*W*_2_: it refers to the weight of the sample after the ignition step (g) at 1000 °C.

The free CaO content (%) was determined using the method described by Abo-El-Enein *et al.*^46^ In this procedure, CaO in the hardened sample (at 1, 3, 28 days) reacts with dry glycerol to form calcium glycerate. A finely ground 0.5 g sample is combined with 40 mL of a glycerol/ethanol mixture (1 : 5 by volume), along with 0.5 g of BaCl_2_ as a catalyst and phenolphthalein as an indicator. The mixture is refluxed on a hot plate for 20 minutes, during which the solution turns pink. While still hot, it is titrated with a standardized alcoholic ammonium acetate solution until the pink color disappears. The solution is reheated, and if the pink color reappears, titration is continued until it remains colorless, confirming that the reaction is complete.

The anti-microbial activity of R, R-0.25Z, R-1Z, R-0.25T and R-1T was measured to investigate the impact of low and high dosages of nano-ZnO and TA on inhibiting the growth of bacteria and fungi. A Disc from each specimen (length × width × thickness of 2.5 × 2.5 × 0.3 cm) was placed in a Malt agar plate (MAP) at 28 ± 1 °C containing *Enterococcus faecalis* bacteria (Gram-positive/ATCC-29212), *Escherichia coli* bacteria (Gram-negative/ATCC-8739) and *Candida albicans* fungi (ATCC 10221), as represented in Fig. 3. In this test, the discs were first immersed in water till the pH reached 7–8 to ensure that the occurring inhibition is due to nano-ZnO or tannic acid, not the alkalinity of cement. According to the National-Committee for Clinical-Laboratory Standards,^47^ the MAPs were prepared by autoclave curing of 30 mL of Sabroud-dextrose-agar medium at 121 °C/15Ibs that solidified with 0.1 mL of bacteria or fungi. Finally, the anti-microbial activity was evaluated by measuring the inhibition-zone around discs according to ASTM D4300-01.^48^

Toxicity Characteristic Leaching Procedure (TCLP, EPA Method 1311)^49^ was employed to assess the release behavior of Zn^2+^ ions and tannic acid from cementitious pastes under acidic conditions at pH ∼ 3–3.5. After 28 days of hydration, a 5 g ground cement sample (R-1Z or R-1T) was individually mixed with 100 mL of acidic extraction solution and agitated at 30 rpm/18 h. This means that the initial concentration of each additive (nano-ZnO or TA) in cement sample is 500 ppm. After extraction, the suspension was filtered to obtain a clear leachate. The resulting filtrate was analyzed using complementary analytical techniques. Zn^2+^ ions concentration was analyzed by Atomic Absorption Spectroscopy (AAS, PerkinElmer 3100-model). The measured Zn^2+^ concentration was evaluated against commonly accepted environmental criteria, where values below approximately 5 ppm indicate effective immobilization within the cementitious matrix.^50^ On the other hand, tannic acid was qualitatively identified using a 0.1% (w/v) FeCl_3_ solution,^51^ while its quantitative determination was carried out using a double-beam UV-Visible spectrophotometer (Shimadzu UV-2700) at its characteristic absorption wavelength of 275 nm,^51,52^ in comparison with a standard solution of pure tannic acid (30 ppm). According to Beer–Lamber law, the concentration of released tannic acid and release % can be calculated *via*eqn (2) and (3). Tannic acid, as a naturally derived and low-toxicity organic compound, is not subject to strict regulatory limits; however, low levels of release are generally regarded as evidence of adequate stability within cement-based matrices.2
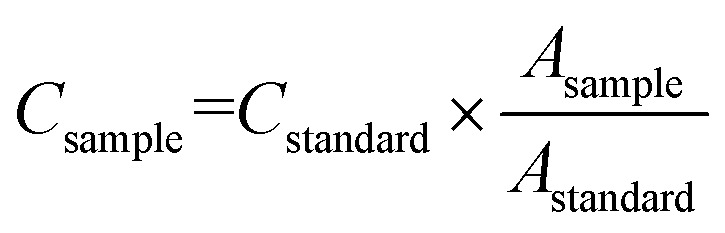
where *C*_sample_ is the released concentration of tannic acid from cement sample, *C*_standard_ donates the standard concentration (30 ppm), and *A*_sample_ and *A*_standard_ correspond to the absorbance values of the released and standard tannic acid solutions, respectively.3
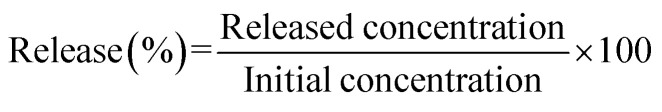


## 3. Results and discussion

### 3.1. Fresh properties

#### 3.1.1. Workability

**Fig. 4:**
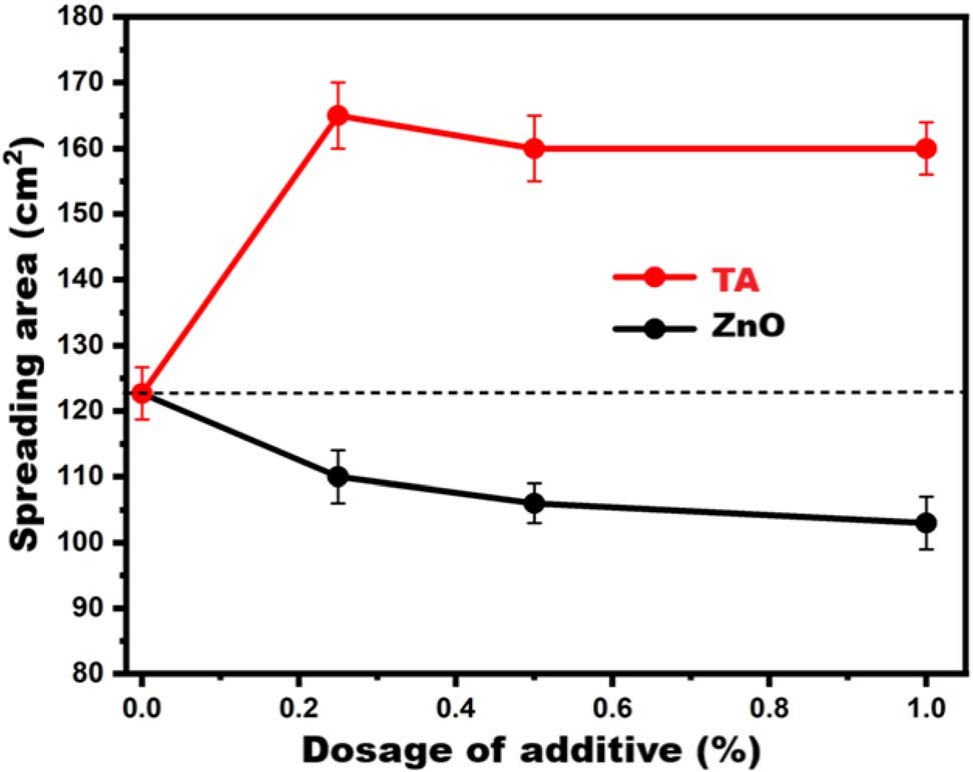
Workability of cement pastes modified with different dosages of nano-ZnO and tannic acid.

Generally, in the mini-slump test, the measured spread-area of the fresh pastes is used to gauge workability; the wider spread-area reflects the high workability.^53–55^Fig. 4 shows a comparison between the impact of nano-ZnO and TA on the spread-area of fresh OPC pastes. For the specimens modified with nano-ZnO, it is noticed a significant reduction in the spread-area compared to the reference one (R); the spread-area decreased by 10.4, 13.6 and 16.1% for R-0.25Z, R-0.5Z and R-1Z, respectively. This finding matched with Pathak and Tiwari.^56^ The high surface-area of nano-particles (nano-ZnO) compared to OPC (29 900 and 3800 cm^2^ g^−1^, respectively) leads to the adsorption of a high amount of mixing water, causing a loss in workability.^57,58^ In the case of TA, the mini-slump test shows a great enhancement in the workability; the spread-area increased by 34.5, 30.4 and 30.4% for R-0.25T, R-0.5T and R-1T, respectively, compared to the reference specimen (R). As illustrated above, TA is a poly-phenolic compound; the reactive terminal phenolic-hydroxyl groups possess a crucial role in its binding to cementitious materials.^26,33,59^ Accordingly, the main reasons for increasing workability after incorporating tannic acid are due to its strong adsorption of particles in the cementitious system, which causes (i) formation of a lubricant film, achieving deflocculation of grains;^33^ (ii) the negatively charged phenolic-oxygen results from the deprotonation of phenolic-hydroxyl groups can disperse the particles through creating an electrostatic repulsion force between them;^59,60^ and (iii) the long side chains of TA prevent agglomeration of cementitious particles through a steric hindrance mechanism.^61,62^ These results fit with Qian *et al.*^34^ and Fang *et al.*^63^ Finally, the reduction in the spread-area with increasing the dosage of TA as in R-0.5T and R-1T with respect to R-0.25 can be attributed to the high molecular weight of TA and its side chain that directs to the pore solution when exceeding the optimum dosages. The presence of TA in the pore solution may hinder the particles' flowability.^64,65^

#### 3.1.2. Setting time

**Fig. 5:**
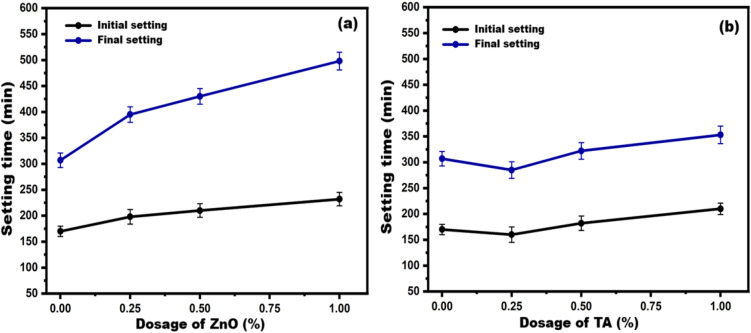
Setting times of cement pastes modified with different dosages of (a) nano-ZnO and (b) tannic acid.

The initial/final-setting time (I/F-ST) values of the reference specimen (R) and others modified with different dosages (0.25, 0.5 and 1 wt%) of nano-ZnO or TA were measured as represented in Fig. 5. Compared to the R specimen, the nano-ZnO shows elongation in the I/F-ST, in line with Mohsen *et al.*^8,15^ This elongation becomes more noticeable with higher dosages; R-0.25Z, R-0.5Z and R-1Z specimens have longer setting times than R by 28, 40 and 62 min for IST and 88, 123 and 191 min for FST, respectively. There are different mechanisms that describe the retardation effect of nano-ZnO. Arliguie and Grandet^66,67^ reported that in the cementitious system, the nano-ZnO present in the pore solution is converted to amorphous Zn(OH)_2_ layers due to the high pH of the pore solution (pH = 12). The formed Zn(OH)_2_ layer will cover the unreacted cement particles (such as tri-calcium silicate phase (C_3_S)), acting as an isolated barrier between such phase and water, preventing the water from reaching it, thus delaying the hydration reaction. On the other hand, Ataie *et al.*^68^ and Garg and White^69^ proposed that the retardation effect of nano-ZnO is due to poising the nucleation site *via* (i) the formation of calcium-zincate-hydrate (CZH) on the nucleation site that hinders the hydration process. CZH may be formed when the Ca^2+^ ions' concentration becomes sufficient in the pore solution; and (ii) adsorption/chelation of Zn^2+^ on the CSH's nuclei, causing blocking of active sites. Furthermore, the chelation of Ca^2+^ ions by Zn^2+^ ions reduces their amount and delays the formation of CSH. On the other hand, the setting behavior of OPC modified with TA was clearly dosage-dependent. The low TA content (0.25 wt%) induced a slight acceleration; the IST and FST were shortened by 10 and 22 min, respectively, concerning the R specimen. Fang *et al.*^61,63^ attributed the acceleration effect of TA to the formation of a considerable amount of Ca-tannate complex, which results from the chelation of Ca^2+^ ions produced from the cement dissolution and TA. This complex enhances the adhesion between the particles in the cementitious system, causing the pastes to lose their plasticity and start setting. Furthermore, Qian *et al.*^34^ reported that the formation of ca-tannate accelerates the hydration process of the OPC, as it acts as a nucleation site, activating the dissolution of cement particles at early ages. In addition to these reasons, 0.25 wt% TA improves workability, enhancing the cement particle's dispersion,^34,63^ accelerating the hydration by increasing the reactive surface area. However, as the TA dosages increased to 0.5 and 1 wt%, the dominant mechanism shifted toward retardation; with respect to the R specimen, the IST was delayed by 12 and 40 min and FST was delayed by 15 and 46 min, respectively. High dosages of TA chelate a large number of Ca^2+^ ions, reducing their availability for hydration product formation at early ages.^29^ Moreover, the TA adsorption onto the cement grains, forming an organic film, prevents their dissolution, thereby retarding the setting.^70^

### 3.2. Hydration kinetics

**Fig. 6:**
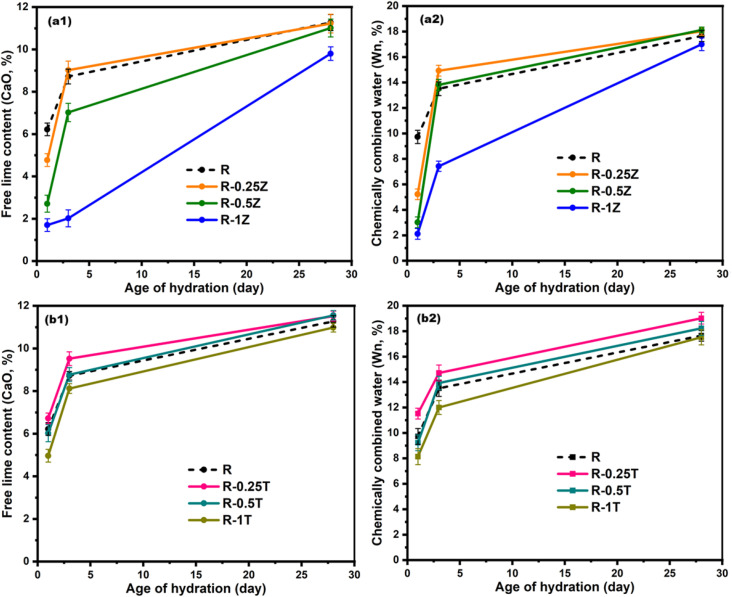
Combined water and free lime contents at different ages of hydration for: (a1 and a2) pastes modified with ZnO NPs and (b1 and b2) pastes modified with tannic acid.

The kinetics of cement hydration can be effectively monitored by assessing both the chemically bound water (*W*_*n*_, %) and the quantity of liberated free lime (CaO, %). Fig. 6-a1 illustrates the evolution of free lime content in the reference paste (R) and in the R-ZnO composite pastes after 1, 3, and 28 days of curing. For the control mixture, the free lime content exhibits a steady increase with hydration time, rising from 6.21% at 1 day to 8.72% at 3 days and reaching 11.27% after 28 days. This gradual rise indicates the sustained formation of calcium hydroxide (CH) resulting from the ongoing hydration of the clinker phases, namely C_3_S, C_2_S, C_3_A, and C_4_AF.^71^ Incorporation of ZnO nanoparticles at various dosages significantly alters the hydration progression, particularly at early curing ages. After 1 day, the free lime content is markedly reduced to 4.72%, 2.71%, and 1.70% for the pastes containing 0.25%, 0.5%, and 1% ZnO NPs, respectively, compared to the control. This pronounced reduction is attributed to the retardation effect induced by the formation of Zn(OH)_2_ layers on the surfaces of cement grains. These hydroxide layers act as physical barriers, limiting ionic diffusion and consequently delaying the onset of OPC hydration reactions. After 3 days of hydration, the free lime content shows differing behavior depending on the ZnO dosage. The paste containing 0.25% ZnO exhibits a slight increase in free lime (9.02%) relative to the reference paste (8.72%), indicating a modest acceleration effect at this dosage. In contrast, the mixes with 0.5% and 1% ZnO display markedly reduced free lime contents of 7.03% and 2.02%, respectively, confirming a stronger retardation response at higher nanoparticle concentrations. Although hydration proceeds as curing time increases, the early-age retardation continues to influence the system. At 28 days, the free lime contents progress to 11.22%, 11.01%, and 9.80% for the respective ZnO-modified pastes, still demonstrating a dosage-dependent reduction relative to the control sample. Overall, these trends confirm that ZnO nanoparticles exert a substantial inhibitory effect on the early hydration of OPC, with higher nanoparticle concentrations producing stronger retardation. Nonetheless, at later ages, recovery of hydration occurs as the Zn(OH)_2_ barrier becomes less effective and transformed into calcium zincate hydrates enabling continued liberation of CH.^18^

The chemically combined water contents of the R, R-0.25ZnO, R-0.5ZnO, and R-1ZnO pastes exhibit a progressive increase throughout the hydration period up to 28 days, as depicted in Fig. 6-a2. The evolution of combined water closely parallels the behavior observed for the free-lime content, where the *W*_*n*_ (%) of the reference paste rises markedly from 9.73% at 1 day to 13.51% at 3 days, eventually attaining 17.65% at 28 days. This continuous growth reflects the gradual accumulations of hydration products such as ettringite (3CaO·Al_2_O_3_·3CaSO_4_·32H_2_O), calcium silicate hydrates, calcium aluminate hydrates, and portlandite.^71^ The introduction of ZnO nanoparticles exerts a pronounced influence on the chemically bound water, most notably at early hydration stages. After 1 day of curing, *W*_*n*_ (%) decreases sharply to 5.12%, 3.22%, and 2.16% for the formulations incorporating 0.25%, 0.5%, and 1% ZnO NPs, respectively, when compared with the reference paste. As hydration proceeds, these values increase to 14.92%, 13.81%, and 7.42% at 3 days and further to 18.02%, 18.12%, and 17.00% at 28 days for the corresponding nanoparticle dosages. These findings underscore the retardation effect imposed by ZnO at early curing ages, followed by a discernible recovery of hydration at later stages as the inhibitory influence of the Zn-rich layers becomes less dominant.^15^

Conversely, varying the dosage of tannic acid (TA) produced distinct effects on the cement hydration process. Incorporating 0.25% TA was adequate to elevate the free lime content from 6.22% to 6.72% and to increase the chemically combined water from 9.72% to 11.52% after one day of hydration, as illustrated in Fig. 6(b1 and b2). This low TA concentration enhanced the hydration kinetics of cement particles by facilitating the formation of moderate amounts of calcium tannate, which served as nucleation centers for hydration product growth and acted as reactive crosslinkers among developing silicate chains.^63^ In contrast, higher TA dosages, up to 1%, exerted a detrimental effect, reducing free lime to 4.97% and chemically bound water to 8.16%. At elevated concentrations, tannic acid chelated significant quantities of calcium ions, thereby hindering the formation of portlandite and other strength-contributing hydrates. Furthermore, excessive TA adsorption around cement grains created isolating layers that temporarily suppressed the hydration reaction. The retarding effect of tannic acid gradually disappeared as hydration advanced, and by 28 days the early inhibitory interactions were fully overcome. As the system stabilized and calcium availability increased, the adsorption layers weakened, allowing hydration to proceed normally. Consequently, long-term hydration products formed without further obstruction.^29^

### 3.3. Compressive strength

**Fig. 7:**
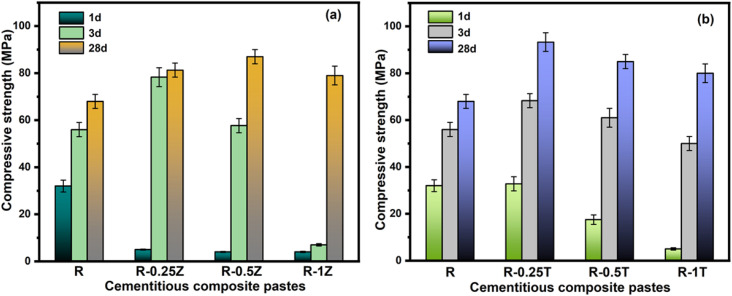
Compressive strength of cement pastes modified with different dosages of (a) nano-ZnO and (b) tannic acid.

To investigate the impact of nano-ZnO and TA on the mechanical properties of the cementitious system, the early and later compressive-strength was measured at 1, 3 and 28 days, as represented in Fig. 7. Generally, for all specimens, a great enhancement in the compressive-strength values is observed with time.^55,72,73^ The accumulation of hydration products (strength-giving-phases), such as calcium-silicate-hydrate (tobermorite, CSH), calcium-alumino-silicate-hydrate (stratlingite, CASH), and calcium-aluminate-hydrate (hydrocalumite and/or hydrogarnet, CAH) fills the pores,^74,75^ increasing gel/space ratio, compacting the microstructure, thus enhancing the compressive-strength.^76–78^

For the specimens containing nano-ZnO (Fig. 7a), R-0.25Z, R-0.5Z and R-1Z show a notable reduction in the early compressive-strength at 1 day; they are lower than the reference specimen (R, 0 wt% nano-ZnO) by 84.4, 87.5 and 87.5%, respectively. These results refer to the detrimental impact of nano-ZnO on the early compressive strength, matching with Mohsen *et al.*,^8^ Ramadan *et al.*^11^ and Abo-El-Enein *et al.*^14^ At 3 days, the R-0.25Z and R-0.5Z specimens exhibit a significant enhancement in the compressive-strength, exceeding the strength of the R specimen by 39.8 and 3.0%, respectively. For the high dosage of nano-ZnO (1 wt%), the specimen still has a low compressive-strength; it is lower than that of the R specimen by 87.5%. This indicates that the regression in compressive strength continues at high nano-ZnO dosages. On the other hand, an opposite trend for all specimens at a later age (28 days) is detected, in line with Mohsen *et al.*^15^ The compressive-strength values of R-0.25Z, R-0.5Z and R-1Z were enhanced by 19.5, 27.9 and 16.2%, respectively, concerning the R specimen. Generally, it can be attributed the low strength value after inclusion of nano-ZnO in the cement matrix to its high retardation effect that hinders the formation of the strength-giving-phases.^15,79^ While the high later compressive-strength may result from that the nano-ZnO particles (i) act as active nucleation sites for the precipitation of strength-giving-phases, promoting the hydration of unreacted particles; (ii) refine the pore structure as they act as filler due to their high surface area and (iii) compact the microstructure through the created CZH phase that formed between pores. All these reasons refer to the formation of a denser microstructure and high mechanical performance.^8,11,80,81^

**Fig. 8:**
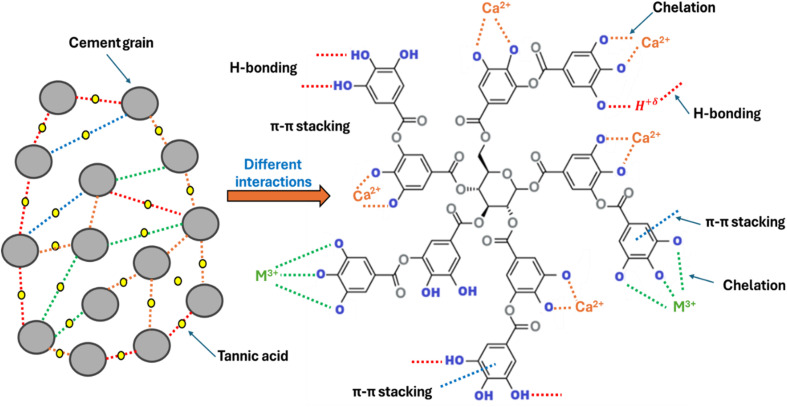
A schematic diagram clarifies the interaction between cementitious components and tannic acid.

Regarding the specimens modified with different dosages of TA (Fig. 7b), at an early age (1 day), it is observed that the R-0.25T has a compressive-strength value close to the R specimen (32.8 and 32 MPa, respectively), on contrast to R-0.25Z, which shows a significant strength reduction. With the increase in TA wt%, a reduction in 1 day compressive-strength is detected. R-0.5T and R-1T specimens have strengths lower than R by 45.3 and 84.4%, respectively, which agrees with Qian *et al.*^34^ As illustrated above, TA has a high tendency to capture Ca^2+^ ions, resulting from the dissolution of cement particles to form calcium-tannate. The formation of such a complex refers to the consumption of most of the Ca^2+^ ions, making them insufficient to produce strength-giving-phases at an early age.^59^ With extending the curing period, R-0.25T, R-0.5T and R-1T show an improvement in the strength values compared to the R-specimen; they increased by 37.21, 25.0 and 17.6% at 28 days, respectively. This improvement may be due to (i) adsorption of tannic acid on the cement matrix component improves cross-linking between hydration products, increasing adhesion; thus reducing the pore and compacting the microstructure;^26,33^ (ii) the enhancement in the dispersion of cement particles after inclusion of TA improves the distribution of formed hydration products in the cement system, refining the pores;^34^ and (iii) the formation of *in situ* calcium-tannate nano-particles, which provides as active seeds for the formation hydration products, simultaneously with acting as a nanofillers, enhancing the particles packing.^28,34,82^Fig. 8 shows a schematic diagram describing the role of TA in enhancing the mechanical performance.

### 3.4. Pore analysis at early age

**Fig. 9:**
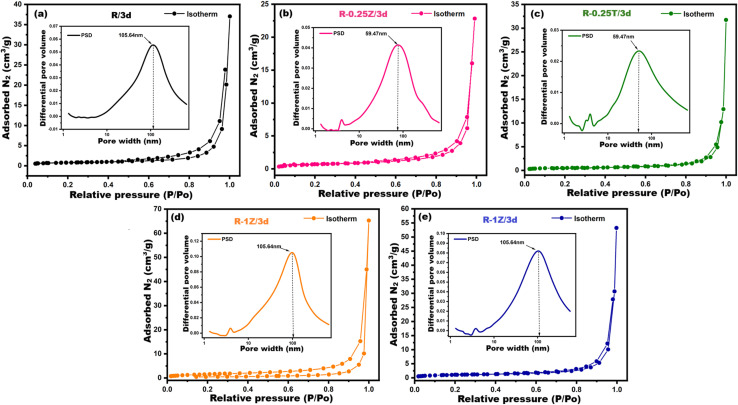
N_2_-adsorption/desorption isotherms and particle size distribution of: (a) R, (b) R-0.25Z, (c) R-0.25T, (d) R-1Z, and (e) R-IT at 3 days of curing.

A comprehensive evaluation of porosity and textural properties at early hydration ages is crucial for interpreting the compressive strength of cement pastes incorporating varying dosages of nano-ZnO and tannic acid. Textural parameters, including BET-specific surface area (SSA, cm^2^ g^−1^), maximum adsorption capacity (AC_max_, cm^3^ g^−1^), total pore volume (*V*_p_, cm^3^ g^−1^), BJH-maximum pore diameter (d*p*_max_, nm), and BJH-average pore diameter (APD, nm), were quantified using the BET and BJH analytical models, as these parameters reliably describe the porous architecture of the fabricated pastes. Fig. 9a–e illustrates the N_2_ adsorption/desorption isotherms and BJH pore size distributions of R, R-0.25Z, R-0.25T, R-1Z, and R-1T pastes cured for 3 days under tap water at room temperature. All investigated pastes exhibit type III adsorption isotherms, indicating a relatively low adsorption capacity. As illustrated in Fig. 9(a–c) and summarized in Table 3, the incorporation of 0.25 wt% ZnO NPs and 0.25 wt% tannic acid resulted in a pronounced reduction in AC_max_ of OPC pastes, decreasing from 37.03 cm^3^ g^−1^ to 22.83 cm^3^ g^−1^ and 31.73 cm^3^ g^−1^, respectively, at *P*/*P*_0_ = 0.99. Furthermore, these low addition levels led to a significant refinement of the pore structure, as evidenced by the shift in d*p*_max_ from 105.64 nm to 59.47 nm and the concomitant decrease APD from 67.92 nm to 45.57 nm and 48.52 nm, respectively. At a dosage of 0.25 wt%, nano-ZnO primarily functions as a nano-filler and heterogeneous nucleation agent, thereby accelerating early-age hydration reactions and promoting the formation of additional hydration products, including CSH, CAH, and CASH phases.^83^ The progressive accumulation of these hydration products contributes to the infilling of capillary pores, leading to reduced pore connectivity and a marked decrease in characteristic pore diameters.^14^ Consequently, nitrogen adsorption at high relative pressures is significantly diminished, as reflected by the lower maximum adsorption capacity, alongside a transformation of coarse pores into mesoporous structures. In the case of tannic acid, pore refinement is primarily associated with its adsorption behavior on OPC particle surfaces, which enhances cross-linking among the newly formed hydration products and promotes microstructural densification.^24^ Moreover, the uniform dispersion of low tannic acid dosages within the cement matrix facilitates the *in situ* formation of calcium-tannate nanoparticles, which serve both as active nucleation sites for hydration products and as nano-scale fillers. This dual role further improves pore rearrangement and contributes to the development of a more compact and refined pore network.^27^

**Table 3:** Texture characteristics for R, R-ZnO, and R-TA pastes at early age of curing

Composite	BET-SSA (cm^2^ g^−1^)	Max-adsorption (cm^3^ g^−1^)	(*V*_p_, cm^3^ g^−1^)	d*p*_max_(nm)	APD (nm)
R	27 120	37.03	0.040	105.64	67.92
R-0.25Z	25 500	22.83	0.035	59.47	45.57
R-0.25T	19 050	31.73	0.027	59.47	48.52
R-1Z	58 880	65.26	0.073	105.64	81.42
R-1T	37 570	53.22	0.056	105.64	54.41

**Fig. 10:**
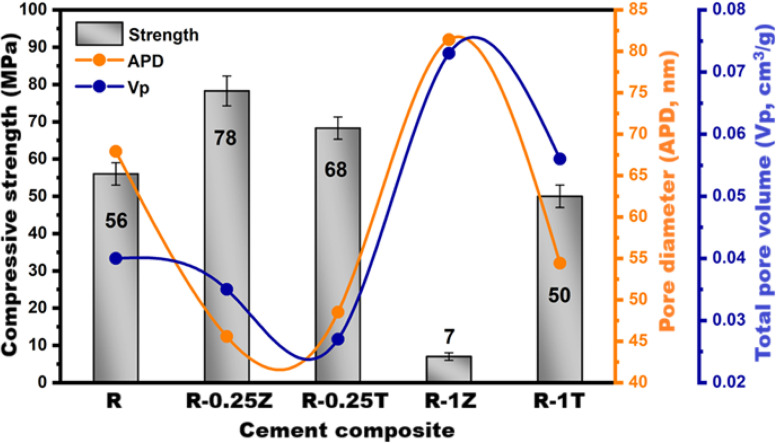
Relation between 3 days-strength and the corresponding texture parameters for some selected hardened pastes.

In contrast, higher dosages of ZnO nanoparticles (1 wt%) and tannic acid (1 wt%) led to a pronounced increase in the maximum adsorption capacity of OPC pastes, rising from 37.03 cm^3^ g^−1^ to 65.26 cm^3^ g^−1^ and 53.22 cm^3^ g^−1^, respectively (Fig. 9d and e). Notably, these elevated concentrations exerted no significant effect on the maximum pore diameter (d*p*_max_); however, they induced substantial modifications in the average pore diameter, which increased from 67.92 nm to 81.42 nm in ZnO-modified pastes, while shifting to 54.41 nm in systems incorporating tannic acid. In addition, the total pore volume increased markedly from 0.040 cm^3^ g^−1^ to 0.073 cm^3^ g^−1^ and 0.056 cm^3^ g^−1^ for ZnO- and TA-modified pastes, respectively. At these higher addition levels, the deleterious effects associated with nanoparticle agglomeration and hydration retardation outweigh the beneficial filler and nucleation roles observed at lower dosages, resulting in the development of a coarser and more open pore structure. Fig. 10 illustrates a clear relationship between the 3 days compressive strength and the average pore size and total pore volume of the prepared pastes. The incorporation of low dosages of ZnO nanoparticles and tannic acid markedly improved early-age strength (from 56 to 78 and 68 MPa) through effective pore refinement. In contrast, higher dosages adversely affected early compressive strength, with the most pronounced reduction observed in ZnO-modified pastes. Although both additives increased porosity at elevated concentrations, the hydration-retarding effect of 1 wt% tannic acid was less severe than that of 1 wt% ZnO. Tannic acid primarily delays hydration through calcium complexation while maintaining uniform dispersion, allowing relatively homogeneous hydration once calcium availability is restored and limiting pore coarsening. Conversely, ZnO nanoparticles at high dosage tend to agglomerate, creating localized regions of inhibited hydration and disrupting continuous hydration product formation, which results in more pronounced pore enlargement (*V*_P_ = 0.073 cm^3^ g^−1^ and APD = 81.42 nm) and greater impairment of microstructural densification (7 MPa).

### 3.5. Phases composition

#### 3.5.1. XRD analysis

**Fig. 11:**
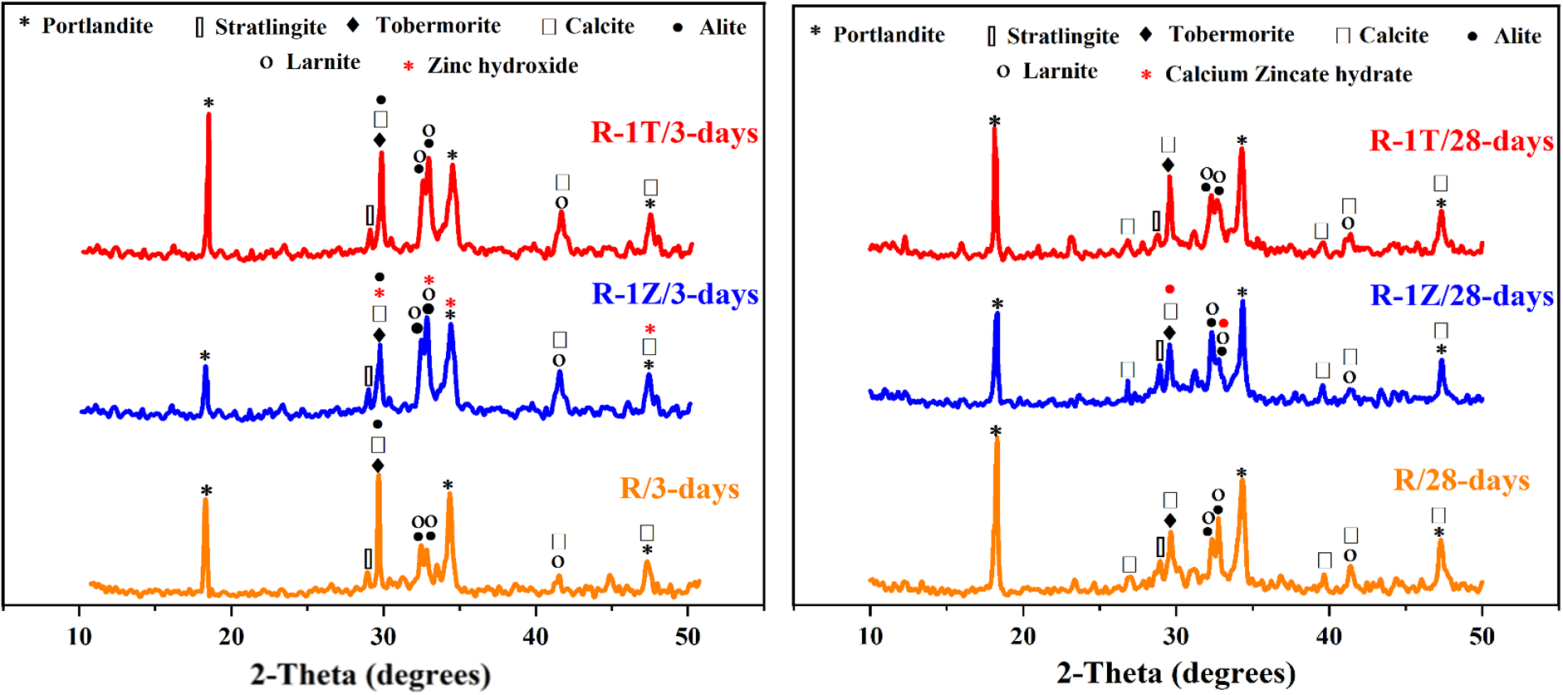
XRD analysis of R, R-1Z and R-1T specimens at 3 and 28 days.

The impact of 1 wt% nano-ZnO or TA on the phase composition of the cement matrix at 3 and 28 days is represented in Fig. 11. At 3 days, the XRD-pattern of the R specimen (100 wt% OPC without nano-ZnO or TA) represents the presence of some distinguishable peaks associated to unreacted phases such as alite (C_3_S, Ca_3_SiO_5_, 2*θ* = 29.69, 32.49 and 32.87°, PDF# 96-154-0706) and larnite (β-C_2_S, Ca_2_SiO_4_, 2*θ* = 32.49, 32.87 and 41.47°, PDF# 96-901-7425). Owing to the hydration process, several peaks related to the hydration product are detected, for instance, portlandite (CH, Ca(OH)_2_, 2*θ* = 18.35, 34.31 and 47.41°, PDF# 96-100-0046) that may be carbonated and transformed to calcite (CC, CaCO_3_, 2*θ* = 29.69, 41.47 and 47.41°, PDF# 96-100-0046). Also, peaked correlated to stratlingite (CASH, Ca_2_Al_2.11_Si_1.11_O_16.25_H_18_, 2*θ* = 29.02°, PDF# 96-900-5060) at and tobermorite (CSH, Ca_2_Si_3_O_11_H_6_, 2*θ* = 29.69°, PDF# 96-900-2247) are observed; these phases are the strength-giving-phases results in strengthening of cement pastes. For the R-1Z/3 days specimen, the XRD-pattern shows the same peaks with different intensities, referring to the significant impact of nano-ZnO on the kinetics of the hydration reactions. Strong peaks related to the unreacted phase (alite and larnite) associated with a lowering in the intensities of the hydration products peaks (portlandite, stratlingite and tobermorite) with respect to the R specimen are detected. This is considered strong evidence of the retardation impact of nano-ZnO and its detrimental impact on early compressive-strength (87.5% lower than R), matching with Mohsen *et al.*^8^ The existence of peaks related to zinc hydroxide (ZH, Zn(OH)_2_, 2*θ* = 29.69, 32.87, 34.31 and 47.41°, PDF# 96-100-0046) is discussed this retardation effect, as illustrated above (acts as an isolated layer). For the R-1T/3 days, an increase in the peaks related to the hydration products is distinguished, mentioning the role of TA in the formation of *in situ* active seeds that nucleate and enhance the dispersion of formed hydration products. Also, this discusses the role of TA in improving the early compressive-strength.

At 28 days, all specimens (R, R-1Z and R-1T) show a decrease in the intensities of peaks related to the unreacted phases and an increase in the intensities of hydration products due to the progression in the hydration process with the curing time. Furthermore, in the case of the R-1Z specimen, the peaks related to zinc hydroxide disappeared and others correlated to calcium-zincate-hydrate (CZH, CaZn_2_(OH)_6_·2H_2_O, 2*θ* = 29.55 and 32.81°) are detected. This observation is matched with Mohsen *et al.*,^8^ when the formation of CZH in the cement matrix at 28 days after inclusion ZnO dust. Disappearance of zinc hydroxide peaks refers to the elimination of the isolated layer (Zn(OH)_2_-layer) from the matrix and continuously in the hydration process. Finally, it can be reported that most of the peaks have comparable intensities, matching the compressive-strength values at 28 days (68, 79 and 80 MPa, for R, R-1Z and R-1T, respectively).

#### 3.5.2. FTIR-spectrum analysis

**Fig. 12:**
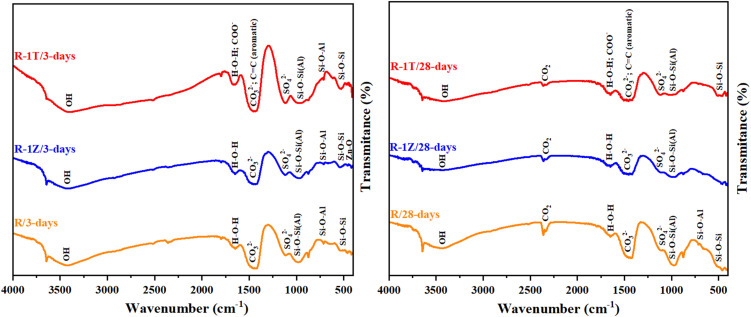
FTIR analysis of R, R-1Z and R-1T specimens at 3 and 28 days.

FTIR-spectrum of R specimen (OPC without additives), R-1Z (OPC + 1wt% nano-ZnO) and R-1T (OPC + 1 wt% TA) at 3 and 28 days are represented in Fig. 12. The spectrum of the three specimens displays multiple distinct transmittance bands at various wavenumbers between 400 and 4000 cm^−1^. At 3 days, the FTIR bands for the R specimen are typically observed in the ranges of (i) 3150–3700 cm^−1^ centered at 3450 cm^−1^ affiliated with O–H stretching of portlandite (Ca(OH)_2_) and physically-adsorbed water, as well as chemically-bound water in the hydration products (CSH and CASH);^84^ (ii) 1590–1740 cm^−1^ centered at 1650 cm^−1^ associated with H–O–H bending of physically-adsorbed/chemically-bound water;^85^ (iii) 1340–1590 cm^−1^ centered at 1440 cm^−1^ related to CO_3_^2−^ asymmetric stretching in calcite (CaCO_3_).^86^ The presence of such a phase indicates that the cement pastes are undergoing carbonation by the reaction of Ca(OH)_2_ with CO_2_ from the air; (iv) 1070–1160 cm^−1^ centered at 1110 cm^−1^ correlated with SO_4_^2−^ asymmetric stretching in gypsum or ettringite;^87^ and (v) 400–1070 cm^−1^, several distinguishable bands result from the formation of CSH and CASH are detected. They are centered at 980, 716 and 540 cm^−1^, allied to Si–O–Si(Al) asymmetric stretching, Si–O–Al symmetric stretching and Si–O–Si bending, respectively.^8^ Generally, the bands assigned to O–H, H–O–H and Si–O–Si(Al) are essential for tracking reaction kinetics.^88,89^ On the other hand, the FTIR spectrum of the R-1Z specimen obviously indicates that the incorporation of 1 wt% retards OPC hydration. Relative to the R specimen, incorporation of nano-ZnO in the matrix leads to (i) the appearance of a new band at 417 cm^−1^, ascribed to the Zn–O stretching vibration, confirming the presence of nano-ZnO;^90^ and (ii) a marked reduction in intensities of OH and Si–O–Si(Al) bands, evidencing the impact of nano-ZnO in inhibiting the hydration of alite/larnite (C_3_S/β-C_2_S) phases and then reducing CSH and CASH yield. This greatly matches the XRD results, in line with Mohsen *et al.*^8^ As illustrated above, the retardation behavior of nano-ZnO can be attributed to the formation of zinc-bearing passivation layers (Zn(OH)_2_) on the OPC's particles, hindering the nucleation and growth of CSH and CASH. Also, these observations explain the drastic reduction in the compressive-strength from 56 MPa (R specimen) to 7 MPa (R-1Z specimen).^8^ In the case of a specimen modified with 1 wt% TA (R-1T), the FTIR evidence points to no meaningful changes in band intensities compared with the R specimen. The only difference is an increase in the intensities of the H–O–H (1590–1740 cm^−1^) and CO_3_^2−^ bands (1340–1590 cm^−1^) due to their overlapping with the COO^−^ asymmetric stretching^91^ and C=C (aromatic) asymmetric stretching,^92^ respectively. The existence of these groups is consistent with phenolic/carboxylate functionality interacting with Ca^2+^, forming Ca-tannate, as proved by Li *et al.*^27^ This indicates no significant delay in the early hydration process at 3 days, as well as it strongly aligns with the nearly identical compressive-strengths (R 56 MPa and R-1T 50 MPa). Moreover, this proves that the formation of Ca-tannate is the main reason behind the superior early mechanical performance. The formation of Ca-tannate adheres the cement particles by increasing the crosslinking between them^61,63^ and acts as a nanofiller and nucleation site for the load-bearing hydrates.^28,34,82^

After 28 days, all specimens present spectral evolution compatible with progression in the hydration process. Generally, it is detected (i) a reduction in the intensity of water bands at 3450 and 1650 cm^−1^, reflecting loss of free water due to its consumption in the hydration process. Also, this reduction refers to refining the pores by forming an excessive amount of hydration products; and (ii) a narrowing and wavenumber upshifting of the Si–O band in Si–O–Si(Al); it is strongly detected in R-1Z and R-1T specimens. The Si–O–Si(Al) shifted from 970 cm^−1^ to 980–990 cm^−1^, which is considered as fingerprint evidence for the progression of hydration of silicate units, and then the formation of CSH. Interestingly, noticing the disappearance of the Zn–O band, referring to the removal of the passive Zn(OH)_2_ layer and continuing the hydration process, matching with XRD results and Mohsen *et al.*^8,15^ Acting nano-ZnO at 28 days as nucleation/filler particles increases the compressive-strength of R-1Z to 79 MPa, exceeding OPC, which has 68 MPa. Regarding TA (R-1T), it leaves early hydration intact and yields modest late-age densification, explaining the continuous enhancement in the mechanical performance, reaching 80 MPa.

### 3.6. Microstructure

The hydration process of cementitious materials is highly sensitive to the presence of chemical and mineral additives.^93^ These additives not only modify the kinetics of hydration but also influence the nature of the hydration products.^94^ The incorporation of various additives significantly influences the type, quantity, and morphology of the resulting hydrates, thereby affecting the microstructure and mechanical properties of cementitious pastes.^95^

**Fig. 13:**
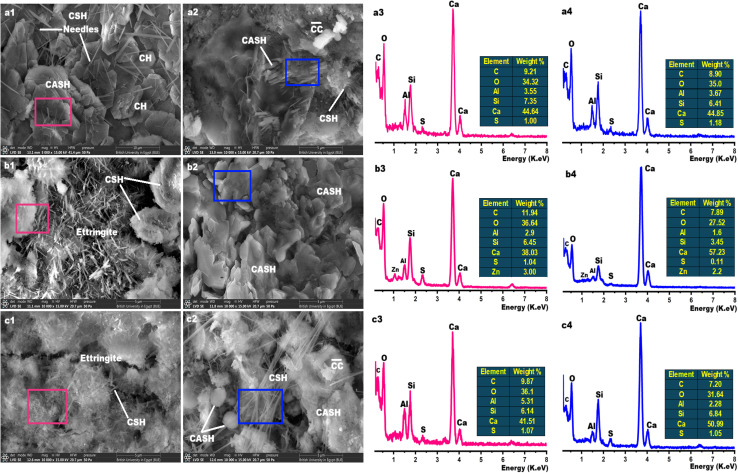
SEM/EDX images of: R (a1–a4), R-1Z (b1–b4), and R-1T (c1–c4) hardened pastes at 3 and 28 days of curing.

Fig. 13 presents SEM/EDX micrographs of the R, R-1Z, and R-1T cementitious pastes at both early and later stages of hydration. After three days of curing, the reference paste (R) exhibited a texture characterized by dispersed needle-like crystals^14^ of calcium silicate hydrate (CSH), interwoven with stacked and layered formations of calcium aluminate silicate hydrate (CASH).^96^ Additionally, a significant presence of hexagonal portlandite (CH) was observed (Fig. 13-a1). As hydration progressed up to 28 days, the microstructure of R paste evolved into a more compact and denser configuration, primarily due to the accumulations of stratlingite-type CASH plates^97^ (Fig. 13-a2). This densification of the microstructure is indicative of enhanced mechanical properties in the R paste over time. The EDX elemental analysis of the reference paste revealed six predominant elements: C, O, Al, Si, Ca, and S. The presence of carbon and sulfur is attributed to the formation of carbonated phases, such as calcium carbonate, as well as sulfate-bearing phases like ettringite (AFt) and monosulfate (AFm).^98^ Notably, the Ca/Si ratio increased from 6.07 at 3 days (Fig. 13-a3) to 6.99 at 28 days (Fig. 13-a4), indicating enhanced calcium incorporation into the hydration products. This shift suggests the development of a less polymerized C-S-H structure, contributing to a denser microstructure. On the other hand, the increase in the Al/Si ratio from 0.48 to 0.57 over the curing period indicates greater aluminum incorporation into the C-A-S-H gel, leading to a more cross-linked and stable structure. This structural evolution may also promote the formation of secondary phases like strätlingite^98^ or hydrogarnet, contributing to microstructural densification.

The incorporation of 1% ZnO nanoparticles into OPC paste (R-1Z) resulted in a noticeable modification of early-age hydration morphology. After 3 days, SEM observations (Fig. 13-b1) showed a reduced presence of characteristic needle-like C-S-H and portlandite phases, along with the formation of ettringite and poorly crystalline zinc-containing products. Elemental analysis (Fig. 13-b3) indicated a decrease in calcium content from 44.64% to 38.03%, accompanied by a slight reduction in the Ca/Si ratio from 6.07 to 5.89. This behavior is associated with the retarding effect of ZnO, which promotes the formation of Zn(OH)_2_ and calcium zincate hydrates on the surfaces of clinker phases, thereby hindering their dissolution and hydration.^11,14,99^ At 28 days (Fig. 13-b2), the retardation effect diminished, leading to the development of more abundant and compact hydration products, including C-S-H, C-A-S-H, and CH. Corresponding EDX results (Fig. 13-b4) revealed an increase in calcium content to 57.23% and a higher Ca/Si ratio. Although EDX provides only semi-quantitative compositional information, these trends may reflect the progression of hydration and the evolution of hydrated phases. Previous report^100^ confirmed that variations in Ca/Si ratio can influence the nanomorphology of C-(A)-S-H; however, in the present work, the observed morphological features are primarily interpreted based on SEM analysis, with EDX data serving as supportive qualitative evidence.

The incorporation of 1% tannic acid into OPC paste (R-1T) did not exhibit any noticeable retardation effect on early hydration. Instead, after 3 days of curing, SEM analysis (Fig. 13-c1) revealed the formation of distinctly crosslinked CSH structures, which played a key role in reducing porosity and enhancing the mechanical strength of the R-1T paste. This behavior is attributed to the interaction between tannic acid and calcium ions during the early hydration phase, leading to the *in situ* formation of calcium tannate nanoparticles. These nanoparticles act as effective nucleation sites/cross-linkers,^27,101^ facilitating the rapid and uniform growth of CSH networks. Compared to the ZnO-modified system (R-1Z), elemental analysis of R-1T (Fig. 13-c3) showed a notable increase in the Ca/Si ratio to 6.76, indicating enhanced calcium incorporation into the hydration products. Over 28 days, the microstructure further evolved, displaying highly crystalline C-S-H in the form of wire-like morphologies (Ca/Si ratio of 7.45), interwoven with fibrillar and foil-like C-A-S[H structures, as shown in Fig. 13(c2 and c4). These morphological developments reflect the continued hydration and structural refinement promoted by tannic acid, contributing to a denser and more mechanically robust cement matrix.^102^

### 3.7. Anti-microbial activity

**Fig. 14:**
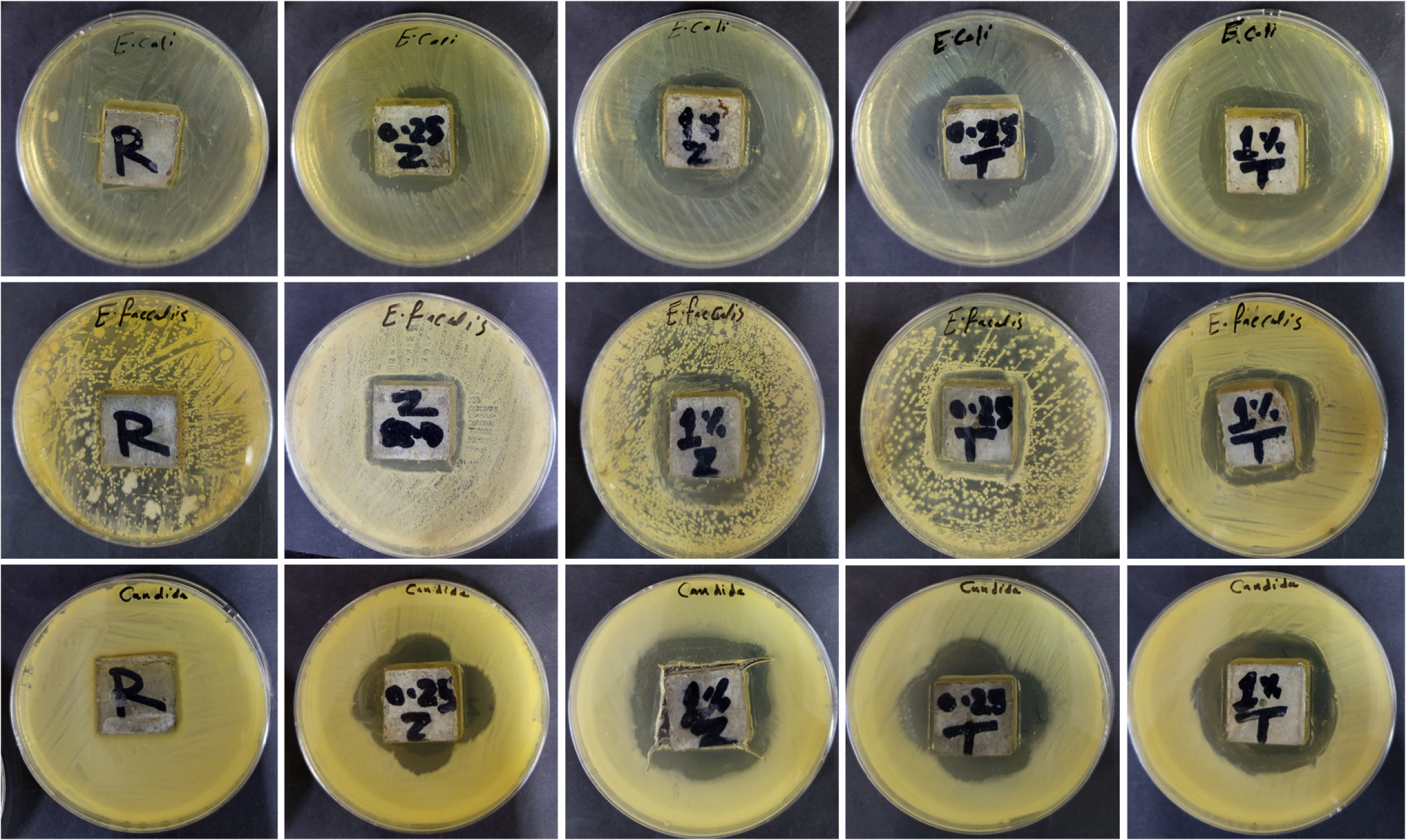
Digital photos for different types of microbial cultures affecting R, R-0.25Z, R-1Z, R-0.25T, and R-1T discs.

To comprehensively evaluate the potential of tannic acid (TA) as a sustainable and multifunctional additive in ordinary Portland cement (OPC), its antimicrobial performance was investigated in addition to its previously demonstrated benefits in enhancing fresh-state properties and mechanical strength. This study compared OPC pastes modified with low (0.25%) and high (1%) concentrations of TA to those incorporating equivalent dosages of nano-zinc oxide (nano-ZnO), a widely used antimicrobial agent. The antimicrobial activity of these formulations was assessed using the agar diffusion method, where unmodified cementitious discs (R), and modified with nano-ZnO (R-0.25Z, R-1Z) or TA (R-0.25T, R-1T) were placed on culture plates inoculated with three clinically relevant pathogenic strains: *Escherichia coli* (ATCC 8739), *Enterococcus faecalis* (ATCC 29212), and *Candida albicans* (ATCC 10221). As presented in Fig. 14 and summarized in Table 4, the unmodified OPC discs (R) exhibited no antimicrobial activity, evidenced by the absence of inhibition zones around the samples. This result highlights the inert nature of neat OPC pastes against microbial colonization. The nano-ZnO-modified specimens (R-0.25Z and R-1Z) exhibited strong antimicrobial activity, underscoring the vital role of nano-ZnO in the advancement of self-cleaning and antimicrobial cementitious materials. The inhibition zone diameters observed around the R-0.25Z discs were 36 ± 1 mm for *Escherichia coli*, 32 ± 1 mm for *Enterococcus faecalis*, and 40 ± 1 mm for *Candida albicans*. When the nano-ZnO concentration was increased to 1%, the inhibition zones expanded significantly to 43 ± 1 mm, 40 ± 1 mm, and 50 ± 1 mm, respectively, indicating a dose-dependent enhancement in antimicrobial performance. The antimicrobial action of nano-ZnO is generally attributed to two primary mechanisms. First, the release of Zn^2+^ ions from the nanoparticles interacts with the negatively charged microbial cell membranes, forming complexes that disrupt osmotic balance. This leads to membrane destabilization, leakage of intracellular contents, and eventual microbial cell death.^15,103,104^ Second, nano-ZnO exhibits photocatalytic properties due to its band-gap energy (∼3.16 eV), enabling it to generate reactive oxygen species under visible or UV light. These induce oxidative stress, damaging cellular structures, altering metabolic pathways, and disrupting DNA integrity, thereby enhancing the antimicrobial effect.^6,11^

**Table 4:** Inhibition zones (mm) measured in various microbial strains

Microbial strain	Cementitious sample				
	R	R-0.25Z	R-1Z	R-0.25T	R-1T
*Escherichia coli*	NA	36 ± 1	43 ± 1	38 ± 1	46 ± 2
*Enterococcus faecalis*	NA	32 ± 1	40 ± 1	34 ± 2	43 ± 1
*Candida albicans*	NA	40 ± 1	50 ± 1	45 ± 0.5	49 ± 2

**Fig. 15:**
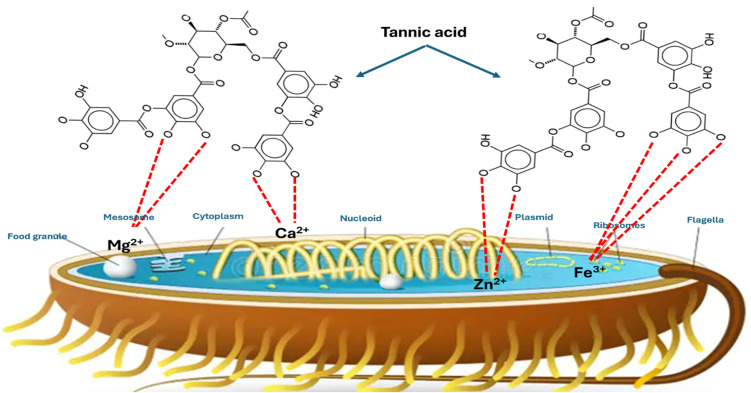
Chelation of tannic acid with some microbial strains.

Cement discs containing tannic acid (R-0.25T and R-1T) exhibited notable antimicrobial efficacy, emphasizing the significant contribution of TA in enhancing the antimicrobial performance cement-based materials. The R-0.25T samples produced inhibition zones measuring 38 ± 1 mm for *Escherichia coli*, 34 ± 2 mm for *Enterococcus faecalis*, and 44 ± 0.5 mm for *Candida albicans*. Increasing the TA content to 1% (R-1T) resulted in a marked enhancement of antimicrobial activity, with inhibition zones expanding to 46 ± 2 mm, 43 ± 1 mm, and 49 ± 2 mm, respectively. The inhibition zone observed in the presence of tannic acid is relatively larger compared to that of nano-zinc, indicating its superior antimicrobial effectiveness. Tannic acid, a naturally occurring polyphenol, exhibits potent antimicrobial activity through multiple synergistic mechanisms. Its antimicrobial efficacy is largely attributed to the presence of numerous hydroxyl groups within its catechol and galloyl moieties, which enable strong interactions (H-bond and dipole–dipole) with microbial cell membranes. These interactions disrupt membrane integrity by increasing permeability, leading to leakage of intracellular contents and eventual cell lysis.^105^ TA also exerts its antimicrobial effect by chelating essential metal ions,^106,107^ such as Fe^2+^ and Zn^2+^, which are vital cofactors for microbial enzymatic systems and metabolic pathways (Fig. 15). This chelation deprives microorganisms of critical nutrients, thereby inhibiting their growth and proliferation.^108^ Additionally, TA can induce oxidative stress by generating reactive oxygen species (ROS), which damage cellular components such as DNA, proteins, and lipids. These multifaceted actions make TA a broad-spectrum antimicrobial agent effective against a wide range of Gram-positive and Gram-negative bacteria, as well as pathogenic fungi.^109,110^ TA is a more sustainable and effective antimicrobial additive for cement-based construction materials compared to nano-zinc oxide. TA, being naturally derived, is environmentally friendly and less toxic.

### 3.8. Leaching behavior

Two cementitious paste formulations, namely (R-1Z) and (R-1T), containing high dosages of additives, were selected to evaluate the immobilization behavior of tannic acid and ZnO NPs. The immobilization behavior of ZnO NPs within the cement paste matrix is evidenced by the extremely low leached concentration of only 0.026 ppm compared to the initial dosage of 500 ppm, corresponding to a negligible release fraction. This value is far below the TCLP regulatory limit for zinc (5 ppm),^49^ confirming the effective stabilization and environmental safety of ZnO NPs in the cementitious system. Such strong immobilization is attributed to multiple complementary mechanisms. Under the highly alkaline conditions of cement paste, ZnO undergoes transformation into insoluble Zn(OH)_2_ and stable calcium zincate hydrates (CaZn_2_(OH)_6_·2H_2_O), as confirmed by XRD analysis. In addition, the nanoparticles are physically encapsulated within the dense and pore-refined cement matrix (R-1Z), which further restricts their mobility and leaching.^8^

**Fig. 16:**
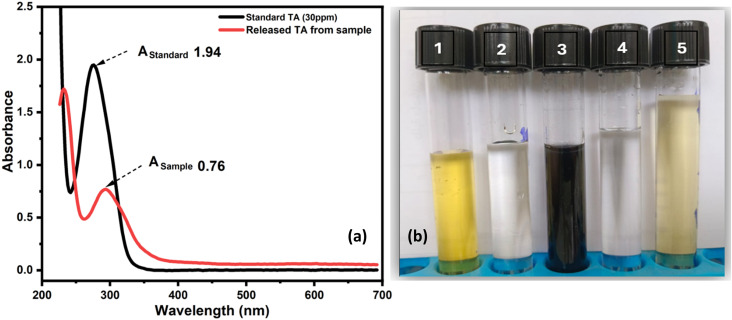
(a) Spectral curves of standard and released tannic acid at *λ*_max_ 275 nm and (b) qualitative analysis for tannic acid using (1) FeCl_3_, (2) standard tannic (30 ppm), (3) Fe-tannate complex, (4) cement filtrate, (5) Fe-filtrate.

The release behavior of TA was evaluated through both qualitative and quantitative approaches using FeCl_3_ complexation and UV-vis spectroscopy, as illustrated in Fig. 16. Qualitatively, the addition of 1 mL FeCl_3_ produces a characteristic dark blue-black color in the presence of TA (tube 3), confirming the formation of the Fe-tannate complex. In contrast, the cement filtrate after the addition of FeCl_3_ (tube 5) exhibits only a faint yellow color due to the addition of FeCl_3_, indicating that negligible complexation occurred and therefore only a very low concentration of free TA is present in solution.^51^ Quantitatively, the UV-vis spectral data at a maximum wavelength of about 275 nm show that the standard 30 ppm TA solution has an absorbance of 1.94, while the released TA from the cement sample shows an absorbance of 0.76. Based on this proportional relationship,^52^ the released TA concentration is approximately 11.8 ppm, corresponding to about 2.36% of the initial 500 ppm tannic acid present in R-1T cement paste. This very low release confirms the strong immobilization behavior of tannic acid in cement paste. This immobilization is primarily driven by chemical interactions, supported by FTIR and XRD analyses, between the phenolic hydroxyl groups of TA and calcium ions, leading to the formation of insoluble calcium-tannate complexes.^29^ In addition, TA exhibits strong adsorption onto cross-linked hydrated gels including tobermorite (CSH) and stratlingite (CASH),^26,33^ along with physical confinement within the dense and refined pore structure of the cement matrix, as evidenced by BET/BJH results. These combined mechanisms greatly reduce the leaching of both TA and ZnO NPs, ensuring their stable retention within the cementitious matrix. As a result, the enhanced antimicrobial performance of R-1Z and R-1T discs is directly supported by the effective immobilization of both additives.

### 3.9. Statistical analysis

To statistically quantify the influence of the key processing variables on the performance of the developed materials, analysis of variance (ANOVA) was conducted. Three main factors were considered in this study: (i) the impact of ZnO wt%; (ii) tannic acid wt%; and (iii) additive type (ZnO and tannic acid) at a constant wt%. Their influence on the fresh properties (workability and final setting time), hardened characteristics (compressive-strength values at 1- and 28 days) and antimicrobial activity (against *Enterococcus faecalis*) were analyzed. From the ANOVA analysis results, several data were obtained, such as sum of squares (SS), degrees of freedom (df), mean square (MS), *F*-statistics (*F*), *F*-critical (*F*-crit) and null hypothesis (*p*-value) with a confidence level of 95% confidence level. The *F*, *F*-crit and *p*-value were used as the key indicators of statistical significance; *F* > *F*-crit and *P* < 0.05 indicate that the parameter exerts a statistically significant effect on the response variable. As presented in Tables 5–7, all investigated factors satisfied the criteria of *F* > *F*-crit and *P* < 0.05, demonstrating that all studied parameters had a statistically significant influence on the measured characteristics^6,11,54,111,112^

**Table 5:** ANOVA results for fresh properties (workability and final setting time)

Factor	Condition	SS		df		MS		*F*	*P*-value	*F*-crit	Significance criteria
		Between groups	Within groups	Between groups	Within groups	Between groups	Within groups				
ZnO wt% on workability	0, 0.25, 0.5, 1 wt%	692.3	130.2	3	8	230.8	16.3	14.2	0.001	4.1	Significant
Tannic wt% on workability	0, 0.25, 0.5, 1 wt%	3455.2	193.1	3	8	1151.7	24.1	47.7	1.89 × 10^−5^	4.1	Significant
ZnO and tannic on workability	1 wt%	5016.3	105.6	2	6	2508.2	17.6	142.5	8.77 × 10^−6^	5.1	Significant
ZnO wt% on final setting	0, 0.25, 0.5, 1 wt%	57 262	2624.7	3	8	19 087.3	328.1	58.2	8.92 × 10^−6^	4.1	Significant
Tannic wt% on final setting	0, 0.25, 0.5, 1 wt%	7250	2870	3	8	2416.7	358.8	6.7	0.014	4.1	Significant
ZnO and tannic on final setting	1 wt%	60 004.7	2039.3	2	6	30 002.3	339.9	88.3	3.55 × 10^−5^	5.1	Significant

**Table 6:** ANOVA results for compressive strength (1 and 28 days)

Factor	Condition	SS		df		MS		*F*	*P*-value	*F*-crit	Significance criteria
		Between groups	Within groups	Between groups	Within groups	Between groups	Within groups				
ZnO wt% on 1 day strength	0, 0.25, 0.5, 1 wt%	1723.8	16.6	3	8	574.6	2.1	277.0	2.03 × 10^−8^	4.1	Significant
Tannic wt% on 1 day strength	0, 0.25, 0.5, 1 wt%	1577.3	44.9	3	8	525.8	5.6	93.7	1.43 × 10^−0.6^	4.1	Significant
ZnO and tannic on 1 day strength	1 wt%	1510.1	17.0	2	6	755.1	2.8	265.8	1.39 × 10^−0.6^	5.1	Significant
ZnO wt% on 28 days strength	0, 0.25, 0.5, 1 wt%	577.7	93.9	3	8	193.6	11.7	16.4	0.0008	4.1	Significant
Tannic wt% on 28 days strength	0, 0.25, 0.5, 1 wt%	1002.6	108.5	3	8	334.2	13.6	24.6	0.0002	4.1	Significant
ZnO and tannic on 28 days strength	1 wt%	269.7	88.9	2	6	134.9	14.8	9.1	0.01	5.1	Significant

**Table 7:** ANOVA results for antimicrobial activity (*Enterococcus faecalis*)

Factor	Condition	SS		df		MS		*F*	*P*-value	*F*-crit	Significance criteria
		Between groups	Within groups	Between groups	Within groups	Between groups	Within groups				
ZnO wt% on antimicrobial activity	0, 0.25, 1 wt%	2691.2	8.8	2	6	1345.6	1.5	916.8	3.47 × 10^−8^	5.1	Significant
Tannic wt% on antimicrobial activity	0, 0.25, 1 wt%	3086	10	3	8	1543	1.7	925.8	3.37 × 10^−8^	5.1	Significant
ZnO and tannic on antimicrobial activity	1 wt%	3460.5	8.8	2	6	1730.2	1.5	1178.8	1.64 × 10^−0.8^	5.1	Significant

## 4. Conclusion

This study demonstrates that tannic acid is a highly effective and sustainable alternative to ZnO NPs for modifying cement paste, offering clear advantages in both fresh and hardened properties. Unlike nano-ZnO, which reduces workability, retards setting, and deteriorates early strength, low dosage of tannic acid enhances paste flowability while maintaining acceptable setting times and promoting strong early-age mechanical performance. The enhanced performance of tannic acid-modified pastes can be attributed to its polyphenolic structure, which promotes improved particle dispersion, accelerates hydration processes, and facilitates microstructural densification through pore refinement and the increased formation of hydration products. These findings are supported by XRD, FTIR, SEM/EDX, and BET/BJH analyses. Collectively, these effects contribute to the development of a denser, more coherent, and mechanically stronger cement matrix at early ages. Furthermore, environmental assessment through TCLP procedures combined with UV-vis spectroscopy confirmed the strong immobilization of both nano-ZnO and tannic acid within the cement pastes. The very low leaching values demonstrate that these additives are securely incorporated into the hydrated structure, thereby reducing potential environmental concerns and improving the long-term stability of the developed materials. Although both additives demonstrated significant antimicrobial activity, enabling their use in self-cleaning and hygienic construction applications, tannic acid exhibited superior overall performance. In addition, the eco-friendly, renewable, and cost-effective nature of tannic acid further supports its adoption as a viable replacement for ZnO NPs in sustainable construction materials, particularly in sensitive environments such as hospitals and healthcare facilities.

## Conflicts of interest

The authors declare that they have no known competing financial interests or personal relationships that could have appeared to influence the work reported in this paper.

## Data Availability

The data supporting the findings of this study are available from the corresponding author upon reasonable request.
